# Transcranial direct current stimulation (tDCS) over the dorso-lateral-prefrontal cortex in combination with exercises for the treatment of individuals with chronic low back pain (STOP-Low Back Pain Trial): study protocol for a randomised controlled trial

**DOI:** 10.1136/bmjopen-2025-111649

**Published:** 2026-03-18

**Authors:** Thomas Pourchet, Stéphane Armand, Cristiano Martins, Simon Barrué-Belou, Stéphane Genevay, Pierre Nicolo

**Affiliations:** 1 Medicine, UNIGE, Geneva, Switzerland; 2 Haute école de santé, HES-SO Genève, Genève, Switzerland; 3 UNIGE, Geneva, Switzerland; 4 Kinesiology Laboratory, HUG, Geneva, Switzerland; 5 Haute école de santé, HES-SO Genève, Vernier, Switzerland; 6 La Tour Hopital Prive SA, Meyrin, Switzerland; 7 Rheumatology, HUG, Geneva, Switzerland

**Keywords:** Back pain, Physical Therapy Modalities, Chronic Pain, PAIN MANAGEMENT

## Abstract

**Introduction:**

Chronic low back pain (CLBP) remains a leading cause of disability worldwide and imposes a substantial personal and socioeconomic burden. Despite exercise being the first-line recommended intervention in clinical guidelines, its efficacy on pain relief remains modest and the hypoalgesia induced by exercise seems to be limited in individuals with musculoskeletal pain. Previous transcranial direct current stimulation (tDCS) studies have mainly targeted the motor cortex, yielding heterogeneous results, underscoring the need to evaluate alternative brain areas. Recently, tDCS studies targeting the dorsolateral prefrontal cortex (DLPFC) may enhance endogenous pain modulation and thereby potentiate the response to exercise. This study aims to compare the effects of adjunctive anodal DLPFC tDCS combined with a standardised exercise programme versus sham tDCS combined with the same exercise programme on pain and function in adults with CLBP.

**Methods and analysis:**

This is a single-centre, triple-blinded, parallel-group randomised controlled trial. 48 participants with CLBP will be randomly assigned to receive either anodal tDCS over the left PFC combined with exercises, or sham tDCS combined with the same exercise programme, over nine sessions during a 3-week period. The primary outcome is the change in the multidimensional impact of CLBP, assessed using the Core Outcome Measures Index, from baseline to postintervention (week 4). Secondary outcomes include pain intensity, disability, psychological factors (fear-avoidance beliefs, catastrophising, anxiety, depression), measured at baseline, postintervention and at 12- and 24-week follow-up. Functional brain connectivity via electroencephalography and neuromuscular function of the erector spinae (flexion–relaxation phenomenon) will be measured at baseline and postintervention.

**Ethics and dissemination:**

This study was approved by the Commission Cantonale d’Ethique de la Recherche sur l’être humain (CCER) in December 2022 (reference number: 2022-D0077). Written informed consent will be obtained from all participants. The results will be disseminated through peer-reviewed publications and presentations at national and international conferences.

**Trial registration number:**

NCT05757609.

**STRENGTHS AND LIMITATIONS OF THIS STUDY**

- The study uses a randomised, sham-controlled, triple-blind design to evaluate the effects of transcranial direct current stimulation combined with an active physiotherapy programme in individuals with chronic low back pain.
- Outcomes are assessed at multiple predefined time points, including immediately postintervention and at 6-month follow-up.
- Standardised intervention protocols and blinded outcome assessment are used to minimise performance and detection bias.
- The protocol includes both clinical and neurophysiological assessments, including electroencephalography and the flexion–relaxation phenomenon.
- Limitations include potential attrition during the 6-month follow-up and restricted generalisability due to the specific inclusion criteria.

## Background

 Low back pain (LBP) is a major public health issue^1^ as it represents the leading cause of years lived with disability.^2^ The average annual direct and indirect costs of LBP range from €2.3 billion to €2.5 billion in high-income countries.^3^ With the ageing of the population, the prevalence of LBP is expected to rise over the next decades, with a projected 843 million prevalent cases in 2050.^4^ LBP is considered chronic after 3 months. Chronic low back pain (CLBP) accounts for 20%–25% of patients following a first acute LBP episode^5^ and generates 70%–90% of the total healthcare costs associated with LBP.^6^

The WHO recommends patient education, physical interventions, psychological interventions, medications and multicomponent interventions for the management of CLBP.^7^ However, these guidelines were developed based on evidence ‘very low’ to ‘low’ certainty. While physical therapies and rehabilitation form the cornerstone of CLBP management in most international guidelines,^8^,^12^ the effectiveness of current treatments appears to be limited. Therefore, the development of novel therapeutic approaches is important for patients.^13^

CLBP is associated with a complex interaction between biological, psychological and social factors. Among the biological factors, neurophysiological mechanisms are involved in nociception and pain perception.^14^ The limited effects of these therapies could be partially explained by their failure to restore the endogenous pain modulation system, specifically the descending inhibitory pathways involved in chronic pain modulation.^15^

Structural and functional changes in brain regions implicated in these pathways, such as the prefrontal cortex (PFC) and anterior cingulate cortex, have been documented in people with CLBP.^16^ Moreover, a recent review reported a decrease in grey matter in the left superior frontal gyrus, which is a part of the PFC.^17^ The PFC mediates antinociceptive effects as the main source of cortical afferents to the periaqueductal grey (PAG). Additionally, PFC may contribute to pain chronification through its corticostriatal projections, potentially depending on dopamine receptor activation.^18^ Thus, the PFC plays a crucial role in the biopsychosocial management of pain, including neuromodulation, exercise, mindfulness and cognitive behavioural therapies and constitutes a promising therapeutic target.^18^

Among the various non-invasive neuromodulation techniques, the evidence supporting the benefits of transcranial direct current stimulation (tDCS) for chronic pain remains to be explored.^19^ Two literature reviews and meta-analyses^20 21^ have assessed the effects of tDCS in patients with CLBP, both concluding that there is no strong evidence to support its use in CLBP management and that clinical relevance remains to be explored. In the meta-analysis by Alwardat *et al*,^20^ which evaluated the effect of anodal tDCS compared with sham stimulation, five studies^22^,^26^ were included for pain intensity analysis, while four studies^22^,^2426^ were pooled for disability assessment. No significant effects favouring tDCS were found for either pain or disability. Since all the studies included in the meta-analysis targeted the primary motor cortex and failed to demonstrate effectiveness, the authors suggested that tDCS could be applied to alternative brain regions, such as the PFC.^20^ Furthermore, it has been shown that tDCS stimulation of the dorsolateral PFC (DLPFC) in healthy individuals increases perfusion in brain regions anatomically connected to the DLPFC and involved in pain perception, including the insular cortex, cingulate cortex and PAG.^27^

Most international guidelines recommend exercise as the first-line treatment for CLBP,^8^,^12^ despite its effects on pain being relatively small and not clinically significant.^28^ While certain types of exercise, such as Pilates, motor control, resistance training or aerobic, have shown greater effectiveness in reducing pain and disability compared with others,^29 30^ the diversity of their mechanisms of action allows therapists to tailor treatment to individual patients.^31^

Exercise may induce a hypoalgesic response, potentially mediated by central nervous system and immune system factors.^32^ For instance, exercise-induced hypoalgesia has been observed in healthy individuals following high-intensity aerobic exercise, with both local and remote effects.^33^ However, evidence supporting exercise-induced hypoalgesia in chronic musculoskeletal pain conditions remains limited.^34^ Nonetheless, exercise appears to exert neurophysiological effects on the descending pain control system through interactions between the opioid and endocannabinoid systems, as well as between the opioid and serotonergic systems.^35^

Building on evidence of prefrontal involvement in descending pain control and the modest efficacy of exercise alone, we will evaluate the clinical efficacy of adding anodal DLPFC tDCS to a standardised exercise programme versus exercise plus sham in CLBP.

### Objectives and hypothesis

The primary objective of this study is to compare the effectiveness of repeated nine sessions of anodal tDCS targeting the DLPFC combined with an intensive exercise programme to nine sessions of sham tDCS combined with the same exercise programme. The comparison will focus on pain intensity and the multidimensional impact of pain in patients with CLBP immediately after the intervention.

The secondary objectives are to assess the effects of repeated sessions of tDCS over the DLPFC combined with an exercise programme on:

- Pain, disability and psychological factors:
  Pain intensity, multidimensional impact of pain, functional disability and psychological outcomes (including fear-avoidance beliefs, catastrophising, anxiety and depression) immediately after the intervention (week 4), with follow-ups at 12 and 24 weeks.
- Functional brain connectivity:
  Changes in functional resting-state brain connectivity as evaluated by electroencephalography (EEG) over the DLPFC and its associated neural networks, between baseline and immediately after the intervention (week 4).
- Neuromuscular function of the erector spinae:
  The restoration of the flexion–relaxation phenomenon (FRP) in the erector spinae during forward bending was assessed immediately after the intervention (week 4).

We hypothesise that anodal tDCS targeting the DLPFC, combined with an intensive exercise programme, will lead to greater improvements in all clinically measured outcomes compared with sham tDCS combined with the same exercise programme, both immediately after the intervention (week 4) and at the 12- and 24-week follow-ups. Regarding functional brain connectivity, we hypothesise an increase in DLPFC connectivity with associated areas, and for the neuromuscular function, a restoration of FRP in the erector spinae muscles. Further, the magnitude of these neurophysiological changes will correlate with clinical improvements.

This protocol is version 1.0, dated 12 February 2026.

## Materials and methods

### Study design

This study is a triple-blind, parallel-group, randomised sham-controlled trial (RCT). The participants will be blinded to their treatment allocation. Blinding will be maintained for the physiotherapists, outcomes evaluators and statisticians.

**Figure 1:**
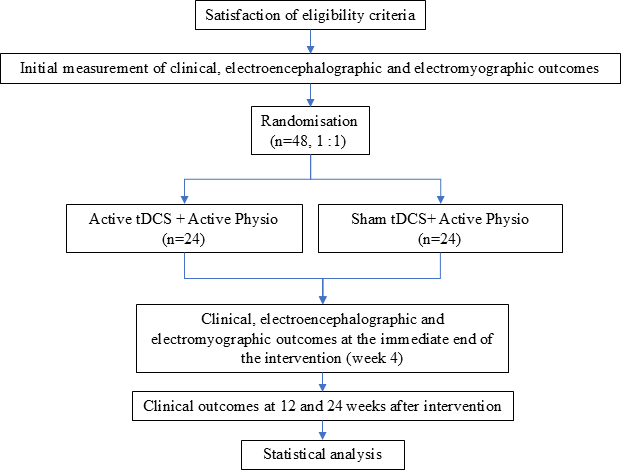
Flow diagram of the study process. tDCS, transcranial direct current stimulation.

The trial will consist of four assessment time points: baseline, 4 weeks (immediately postintervention), 12 weeks and 24 weeks of follow-up. The intervention will include nine treatment sessions over a 3-week period (figure 1).

During the enrolment visit, eligible participants will complete self-administered questionnaires assessing socio-demographic characteristics.

During the baseline visit, they will complete questionnaires assessing pain intensity, multidimensional pain impact, disability and psychological factors (including fear-avoidance beliefs, catastrophising, anxiety and depression). Then, they will perform surface electromyography (sEMG) to assess the FRP. Finally, they will undergo a resting-state EEG to evaluate functional brain connectivity.

The study will be conducted at the La Tour Hospital in Meyrin. This RCT is registered on ClinicalTrials.gov (NCT05757609) and will follow the Consolidated Standards of Reporting Trials Standard Protocol Items: Recommendations for Interventional Trials (SPIRIT) guidelines.^36^ The completed SPIRIT checklist is provided as online supplemental material 1 for this protocol.

### Participants

The study will include two groups of 24 adult participants, aged 18–65 years, all diagnosed with CLBP, defined as pain localised in the lower back, with or without radiating leg pain, persisting for more than 3 months. Potential participants will be recruited through various networks, including social media campaigns, referrals from physicians or physiotherapists and word of mouth. To be eligible, participants must report an average pain intensity of at least 3 out of 10 on the pain numerical rating scale (NRS) over the past week, with 0 representing ‘no pain’ and 10 representing ‘worst pain imaginable’. Additionally, they must have sufficient cognitive abilities to complete the study questionnaires, demonstrated by a minimum B2 level in French. Exclusion criteria include neuropathic pain (DN4 score ≥4),^37^ radiating pain beyond the knee, sensory or motor deficits of the lower limb, a specific cause of low back pain (eg, inflammatory rheumatic disease or fibromyalgia), lumbar surgery involving instrumentation, herniectomy within the previous 6 months, major neurological or psychiatric disorders, pregnancy and any contraindication to tDCS (eg, epilepsy, pacemaker, metal implants in the skull or skin lesions at electrode sites).

No specific contraindications to exercise were applied beyond the general eligibility criteria. A complete list of inclusion and exclusion criteria is provided in online supplemental material 2.

### Randomisation/blinding

A randomisation list (using permuted blocks of randomly varying sizes 4, 6 or 8) will be generated by an independent research assistant using a computer-generated random number system (https://www.sealedenvelope.com/). To ensure allocation, consecutively numbered, sealed opaque envelopes will be used. Participants will be randomly assigned in a 1:1 ratio to receive either an anodal tDCS combined with an exercise programme or a sham tDCS combined with the same exercise programme. Patient-reported outcomes will be completed by participants themselves through an online procedure using Research Electronic Data Capture (REDCap), a secure, web-based platform designed to facilitate data collection in research studies.^38^ This system ensures that outcome assessors remain blinded, preventing any potential bias in data collection.

Neurophysiological and neuromuscular outcomes will be collected by trained assessors who are not involved in treatment delivery and who are blinded to group allocation.

The researchers responsible for data analysis will be blinded to treatment allocation. Similarly, all outcomes’ assessors and statisticians will remain blinded.

Both participants and the physiotherapist administering the exercise programme and the application of tDCS will also be blinded to treatment allocation. To ensure blinding, an independent research assistant will have previously programmed the *double-blind mode* available in the device’s software interface (DC-stimulator; Soterix Medical, New York, USA). This mode allows the system to automatically generate either an active or sham stimulation after the parameters for both options have been configured. The system can be operated in a special password-protected mode, ensuring that the information displayed on the screen does not reveal whether the stimulation is active or sham. The research assistant will assign a unique session-specific code to be provided to the physiotherapist before each session. Emergency unblinding will only be conducted in the event of a serious adverse event that is both unexpected and suspected to be related to the intervention.

### Interventions

#### Transcranial direct current stimulation

Previous studies have shown that anodal stimulation of the PFC may be a promising approach for pain relief,^20 27^ likely due to the activation of the descending pain modulation pathways. Therefore, a tDCS device, DC-stimulator (Soterix Medical) will be applied by the physiotherapist during the aerobic part (cycling) of the exercise programme. The anode (positive excitatory electrode) will be placed over the left PFC and the cathode (negative electrode) over the right supraorbital area. The stimulation will be delivered for 20 min using a continuous current at an intensity of 2 mA through two electrodes (surface 5×5 cm=25 cm^2^). This protocol aligns with current tDCS guidelines for pain relief^39^ and remains within safety margins.^40^

#### Sham tDCS

Sham tDCS will be administered during the aerobic component of the intervention (cycling), following the same electrode placement as in the active tDCS condition. The same tDCS device is used for both the active and sham conditions, with different pre-programmed modes. On initiation of the session by the physiotherapist via entry of a session-specific code, the device will automatically activate the sham mode. In this mode, the electrical current will gradually ramp up over 30 s and then progressively decrease until it is turned off. This procedure is designed to mimic the initial cutaneous sensations of active stimulation, thereby preserving participant blinding, without producing any significant neurophysiological effects.^41^ The sham stimulation will be delivered for 20 min.

#### Exercises

During each of the nine physiotherapy sessions, participants will complete 20 min of aerobic exercise (cycling). The cycling session will begin with a 5-min warm-up, followed by 10 min at high intensity (corresponding to a perceived exertion of 6–8 on the 0–10 modified Borg-CR10 scale),^42^ and conclude with a 5-min cool-down. Subsequently, participants will engage in a 20-min strengthening circuit targeting the back, abdominal, upper and lower limb muscles. Each session will end with a 10-min global mobilisation exercise involving movements in multiple directions. Including rest periods, the total session duration will be 60 min. The exercise programme is individualised and adapted according to each participant’s physical capacity and pain response.

Exercise intensity and progression are adjusted by the physiotherapist throughout the intervention to ensure safety and tolerability while maintaining a standardised treatment framework. Details of the exercise protocols are available in online supplemental material 3.

### Outcomes

Study outcome measures are presented in table 1 according to the SPIRIT guidelines^36^ and include validated patient-reported measures of pain intensity and physical function, psychosocial questionnaires, neurophysiological and neuromuscular outcomes. Detailed descriptions of each outcome measure and scale are provided in the Outcomes section.

**Table 1:** Standard Protocol Items: Recommendations for Interventional Trials diagram of enrolment, interventions and assessments for the Stimulation to Optimize Physiotherapy (STOP) - Low Back Pain Trial

	Study period						
	Enrolment	Baseline	First-treatment				Follow-up
Timepoint**	−t_1_	0	w1	w2	w3	w4	w12 and w24
Enrolment							
Eligibility screen	X						
Informed consent	X						
	X						
Treatment allocation		X					
Interventions							
tDCS+AP (n=24)			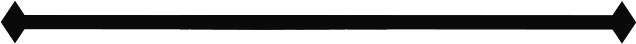				
Sham tDCS+AP (n=24)			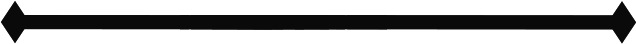				
Assessments							
Baseline demographic	X						
COMI		X				X	X
PNRS		X				X	X
ODI		X				X	X
HADS, FABQ PCS		X				X	X
Rest EEG		X				X	
sEMG FRP		X				X	

0, starting day of the treatment; AP, active physiotherapy; COMI, Core Outcome Measure Index; EEG, electroencephalogram; FABQ, Fear Avoidance Belief Questionnaire; FRP, flexion–relaxation phenomena; HADS, Hospital Anxiety and Depression Scale; ODI, Oswestry Disability Index; PCS, Pain Catastrophising Scale; PNRS, pain numerical rating scale; sEMG, surface electromyography; tDCS, transcranial direct current stimulation; w, weeks.

#### Primary outcome

The primary outcome will be the mean difference in the multidimensional impact of CLBP, as measured by the Core Outcome Measures Index (COMI), between baseline and immediately postintervention (week 4).

The COMI consists of seven items assessing five dimensions: pain, function, symptoms-specific well-being, quality of life and disability. COMI is reliable and has been validated in French.^43^ Scores range from 0 (excellent health status) to 10 (worst health status).

#### Secondary outcomes

The clinical secondary outcomes will be the difference between baseline measures, postintervention measures (week 4) and follow-up measures (weeks 12 and 24) for the:

- Multidimensional impact of CLBP measures by COMI.
- Functional disability measured by Oswestry Disability Index (ODI).
- Mean intensity of pain of 7 last days measured by NRS.
- Psychological impact of CLBP measured by:
  - Fear Avoidance Belief Questionnaire (FABQ).
  - Pain Catastrophising Scale (PCS).
  - Hospital Anxiety and Depression Scale (HADS).

#### Neurophysiological outcome

Spontaneous activity in a task-free state will be assessed using a 10-min resting-state EEG.

EEG data will be continuously acquired at 512 Hz using an Active II EEG system (Biosemi, Amsterdam) with 128 pre-amplified (active) EEG channels with standard 10–20 configuration. Participants will be asked to keep their eyes closed and remain relaxed yet awake. Epochs showing artefacts or reduced vigilance were removed following visual inspection. From the remaining data, 5 min free of artefacts were re-referenced to the common average. Source-level functional connectivity (FC) will be computed in MATLAB (The MathWorks, Natick, Massachusetts, USA) using the open-source NUTMEG toolbox and its Functional Connectivity Mapping module, as previously described. Lead fields on a 10 mm grid will be generated with a boundary element model derived from an individual template 3D T1-weighted MRI (Helsinki BEM Library) via the NUTMEG plugin. Artifact-free EEG epochs will be band-pass filtered between 1 and 20 Hz using a zero-phase elliptic filter and projected to grey-matter voxels with an adaptive spatial filter (scalar minimum-variance beamformer).

Connectivity metrics will be computed separately in seven canonical bands—delta (1–3 Hz), low theta (4–5 Hz), high theta (6–7 Hz), low alpha (8–10 Hz), high alpha (11–12 Hz), low beta (13–16 Hz) and high beta (17–20 Hz). Because between-subject differences in synchronisation magnitude can reflect variations in signal-to-noise ratio, we will normalise each participant’s connectivity map within band by subtracting the subject’s mean Weighted Node Degree (WND) across all cortical voxels and dividing by the corresponding SD, yielding voxelwise z-score maps. The z-maps will then be spatially normalised to canonical MNI space using SPM8 functions (http://www.fil.ion.ucl.ac.uk/spm/software/spm8/).

Relationships between the clinical variables and NIBS-induced changes in effective connectivity/FC will be analysed with Pearson correlations and corrected with a false discovery rate of 5%.

#### Neuromuscular outcome

The FRP corresponds to a myoelectric silence of erector spinae during maximal forward bending. The FRP is reduced or abolished in approximately half of individuals with LBP.^44^

It will be assessed at baseline and post-intervention (week 4) to evaluate potential restoration. A sEMG system (model Trigno, Delsys, Boston, Massachusetts, USA) will be used with a sampling frequency of 1000 Hz. The skin at the electrode sites will be shaved, abraded and cleaned with alcohol, prior to data collection. Electrodes will be placed over the left and right longissimus dorsi (at the level of the L1 spinous process) and the left and right multifidus muscles (at the L5 level) aligned with the orientation of the muscle fibres and according to the recommendations of the Surface Electromyography for Non-Invasive Assessment of Muscles projects.^45^ Standardised instructions will be provided to all participants to ensure consistency in movement execution. Participants will be instructed to stand upright with their arms relaxed at their sides, eyes directed forward and level. They will then be asked to bend forward into maximal trunk flexion while maintaining their knees fully extended, allowing the upper body and arms to hang freely in a relaxed manner.

The movement sequence will be divided into four distinct phases, each lasting five seconds^46^: (1) static upright standing, (2) forward bending into full flexion, (3) static maintenance of full flexion and (4) return to the initial upright standing posture. Specifically, participants will first maintain a neutral standing position for 5 s, followed by a controlled descent into maximal forward flexion over 5 s. They will then hold the fully flexed position statically for an additional 5 s before returning to the starting posture over the final 5 s.

To ensure correct execution, an experimenter will demonstrate the movement sequence and pacing prior to the trials. A metronome will be used to regulate timing and promote temporal consistency across participants. Each participant will perform three consecutive trials per set, and the entire sequence will be repeated twice.

### Sample size calculation

An a priori sample size calculation was conducted using G*Power software (V.3.1.9.4). The estimation was based on the Minimal Clinically Important Improvement for the COMI, considered the primary outcome measure, with a reference value of 2.6 points.^47^ This endpoint was defined as the difference in COMI scores between baseline and immediately postintervention (week 4).

The expected SD of the COMI score in both groups was conservatively estimated at 3, based on a previous study^48^ and expert clinical judgement. With a significance level (α) of 0.05 (two-tailed) and a desired statistical power of 80% (1−β), the required sample size was calculated to be 22 participants per group to detect a clinically meaningful between-group difference.

To account for an anticipated dropout rate of 10%, the recruitment target was adjusted to 24 participants per group. Therefore, a total of 48 participants will be enrolled in this trial.

### Statistical analysis

All primary and secondary outcome measures will be analysed according to the intention-to-treat principle. A two-tailed superiority testing approach will be applied, with the level of statistical significance set at α=0.05 (p<0.05).

#### Primary analysis

To evaluate between-group differences in the primary outcome, the change in COMI score from baseline to immediately postintervention (week 4), an independent samples t-test will be used, provided the data meet the assumption of normality. If the assumption of normality is violated, as determined by appropriate normality tests (eg, Shapiro-Wilk), a non-parametric alternative, the Wilcoxon-Mann-Whitney U test, will be employed to assess the statistical significance of the observed differences.

#### Secondary analysis

The sample size calculation was based on the primary outcome, and the main conclusions of the study will be drawn from this analysis. Secondary outcomes will be considered exploratory.

To address the issue of multiple comparisons, statistical correction procedures will be applied as appropriate. In multivariable models, the commonly used rule of thumb of one predictor variable per ten participants will be followed to reduce the risk of overfitting.^49^

#### Clinical outcomes

The longitudinal evolution of each secondary outcome (COMI, ODI, VAS, HADS, FABQ, PCS) at weeks 4, 12 and 24 will be compared between groups using a mixed-effects regression model.

A random effect for subjects and fixed effects for time and group will be included in the model. To assess potential differences in outcome trajectories between groups over time, an interaction term between time and group will be specified and evaluated using a likelihood ratio test. Intention-to-treat approach will be applied.

#### Neurophysiological outcome

To determine neurophysiological differences between treatment groups (stimulation vs sham), unpaired pseudo-t-tests or their non-parametric equivalents will be conducted, depending on the distribution of the EEG variables of interest. EEG parameters will be analysed both on a voxelwise basis and within predefined regions of interest, with or without correction for multiple comparisons.

#### Neuromuscular outcome (FRP)

The root mean square (RMS) of the EMG signals will be calculated using a sliding window of 1 s with a step size of 50 ms.^50^

The three phases of the flexion-extension cycle—flexion, relaxation and extension—will be manually identified on the RMS signal, and the peak value for each phase will be recorded.

The flexion–relaxation ratio (FRR) will be computed by dividing the peak EMG activity during the flexion phase by that of the relaxation phase. Preintervention and postintervention FRR values will be compared using a paired t-test to assess statistical significance. If the assumption of normality is not met, the non-parametric Wilcoxon signed-rank test will be applied instead.

### Trial monitoring and interim analyses

Given the low-risk nature of the interventions and the short duration of the trial, no data monitoring committee was established. No interim analyses or stopping guidelines were planned. Trial conduct and data integrity are overseen by the investigators.

### Ethics and dissemination

Study data will be collected and managed using REDCap electronic data capture tools, hosted at the University of Applied Sciences and Arts Western Switzerland (HES-SO). REDCap is a secure, web-based software platform designed to support data collection and management for research purposes.^38 51^ This study received ethical approval from the Cantonal Research Ethics Commission for Human Research (Commission Cantonale d’Éthique de la Recherche sur l’être humain—CCER) in December 2022 (reference number: 2022-D0077). Any amendments to the study protocol will be submitted for approval to the ethics committee and updated on ClinicalTrials.gov accordingly. At the baseline visit, all participants will provide written informed consent prior to the initiation of any study-related procedures. Participants will be clearly informed of their right to withdraw from the study at any time, without penalty or loss of benefits. After the inclusion visit, eligible participants will be given a 48-hour reflection period before providing written informed consent. A copy of the participant information and consent form is provided as online supplemental material 4. The results of the randomised controlled trial will be published in a peer-reviewed scientific journal, and de-identified data will be made available on a public repository at the time of publication. Study findings will also be disseminated through presentations at national and international scientific conferences.

### Participants’ withdrawal and adverse events

To minimise safety risks related to the tDCS intervention, the device will be used in accordance with the manufacturer’s instructions. Safety guidelines will be followed, and known contraindications to tDCS will be included as non-inclusion criteria.^40^ The safety of the medical devices will be monitored throughout the study by assessing adverse device effects, serious adverse device effects and serious adverse events. The following information will be recorded in the electronic Case Report Form during the entire duration of the study: onset of the event, duration, resolution, actions taken, assessment of intensity and relationship to the study treatment.

In this study, participants will be invited to report any adverse events either spontaneously or in response to a general, non-directed question (eg, ‘How has your health been since your last visit?’).

If an event is reported, the participant will provide details on the type of event, time of onset, duration, intensity and resolution. An investigation procedure will then be initiated to assess the relationship with the medical device, the expectedness of the event and its severity.

Participants will be free to withdraw from the study at any time, without providing any justification.

### Timeline and feasibility

Participant recruitment began in August 2023 and is expected to be completed by February 2026. The anticipated recruitment rate is approximately two participants per month. Funding for the study has been secured through multiple institutions, including the Geneva School of Health Sciences, the Geneva University Hospitals, the University of Geneva and La Tour Hospital.

## Discussion

This randomised controlled trial is, to date, the first to investigate the effects of repeated sessions combining physical exercises with anodal tDCS targeting the PFC in individuals with CLBP. Previous studies involving tDCS in CLBP have primarily targeted the motor cortex.^20^ Exercise is currently the most widely recommended intervention for CLBP according to international clinical guidelines^52^ and its effects are thought to be partially explained by neurophysiological mechanisms.

While exercise-induced hypoalgesia is well-documented in healthy individuals, this mechanism appears to be attenuated or altered in patients with musculoskeletal pain conditions.^34 53^ We hypothesised that applying anodal tDCS over the PFC during exercise may enhance descending pain inhibition pathways and result in clinically meaningful improvements in both pain and disability among patients with CLBP.

In addition, the inclusion of neurophysiological (EEG) and neuromuscular (FRP) outcome measures may help elucidate the underlying mechanisms of action. This study has several strengths. First, it is designed to provide evidence regarding the efficacy of combining tDCS with exercise. Second, the use of sham tDCS and the implementation of robust blinding procedures allow us to control for sham effects and isolate the specific effects of tDCS.

However, one limitation of this study is that it will be conducted at a single research centre, which may restrict the generalisability of the findings. In addition, adverse events related to tDCS will be collected using standard monitoring procedures rather than a dedicated, standardised questionnaire, which may lead to under-reporting of mild or transient adverse effects.^54^

Despite these limitations, the present trial offers a unique opportunity to evaluate the efficacy of this therapeutic combination using a rigorous and controlled protocol. tDCS is a non-invasive neuromodulation technique that is already widely used in the management of other conditions, such as depression^55^ and has shown potential for implementation in home-based programmes with remote supervision.^56^ If the intervention proves to be effective, it could be translated into clinical practice to improve pain and functional outcomes in individuals with CLBP, thereby helping to reduce the associated social and economic burden.

## Supplementary material

10.1136/bmjopen-2025-111649 online supplemental file 1

10.1136/bmjopen-2025-111649 online supplemental file 2

10.1136/bmjopen-2025-111649 online supplemental file 3

10.1136/bmjopen-2025-111649 online supplemental file 4
