# Supplementary material for: Transcranial direct current stimulation (tDCS) over the dorso-lateral-prefrontal cortex in combination with exercises for the treatment of individuals with chronic low back pain (STOP-Low Back Pain Trial): study protocol for a randomised controlled trial
Source: BMJ Open. 2026 Mar 18;16(3):e111649. doi: 10.1136/bmjopen-2025-111649 (PMC13007130; doi:10.1136/bmjopen-2025-111649)
Supplement: online supplemental file 1 [file bmjopen-16-3-s001.docx]

**Supplementary Material 1: SPIRIT 2025 Checklist**

**SPIRIT 2025 checklist of items to address in a randomized trial protocol***

| **Section / Topic** | **No** | **SPIRIT 2025 checklist item description** | **Reported on page no.** |
| --- | --- | --- | --- |
| **Administrative information** | | |  |
| Title and structured summary | 1a | Title stating the trial design, population, and interventions, with identification as a protocol | p.1 lines 1-3 |
|  | 1b | Structured summary of trial design and methods, including items from the World Health Organization Trial Registration Data Set | **Abstract**, p.2-3, lines 51-83 |
| Protocol version | 2 | Version date and identifier | **Introduction**, p.6, line 174 |
| Roles and responsibilities | 3a | Names, affiliations, and roles of protocol contributors | p.1, lines 5- 33; p.17-18, lines 518-524 |
|  | 3b | Name and contact information for the trial sponsor | p.16-17, lines 500-504 |
|  | 3c | Role of trial sponsor and funders in design, conduct, analysis, and reporting of trial; including any authority over these activities | p.16-17, lines 500-504 |
|  | 3d | Composition, roles, and responsibilities of the coordinating site, steering committee, endpoint adjudication committee, data management team, and other individuals or groups overseeing the trial, if applicable | Not Applicable |
| **Open science** | | |  |
| Trial registration | 4 | Name of trial registry, identifying number (with URL), and date of registration. If not yet registered, name of intended registry | **Materials and methods**, p.7, lines 194-195 |
| Protocol and statistical analysis plan | 5 | Where the trial protocol and statistical analysis plan can be accessed | **Materials and methods** |
| Data sharing | 6 | Where and how the individual de-identified participant data (including data dictionary), statistical code, and any other materials will be accessible | **Materials and methods**, p.15, lines 437-439 |
| Funding and conflicts of interest | 7a | Sources of funding and other support (e.g., supply of drugs) | p.17, lines 499-504 |
|  | 7b | Financial and other conflicts of interest for principal investigators and steering committee members | p.17, lines 506-507 |
| Dissemination policy | 8 | Plans to communicate trial results to participants, healthcare professionals, the public, and other relevant groups (e.g., reporting in trial registry, plain language summary, publication) | p.15, lines 437-440 |
| **Introduction** | | |  |
| Background and rationale | 9a | Scientific background and rationale, including summary of relevant studies (published and unpublished) examining benefits and harms for each intervention | **Background**, p.3-6 |
|  | 9b | Explanation for choice of comparator | **Materiel and methods**, p.9 lines 261-263 |
| Objectives | 10 | Specific objectives related to benefits and harms | Background p.6, lines 166-172 |
| **Methods: Patient and public involvement, trial design** | | |  |
| Patient and public involvement | 11 | Details of, or plans for, patient or public involvement in the design, conduct, and reporting of the trial | p.17, lines 509-511 |
| Trial design | 12 | Description of trial design including type of trial (e.g., parallel group, crossover), allocation ratio, and framework (e.g., superiority, equivalence, non-inferiority, exploratory) | **Materials and Methods** (Study design), p.6-7 |
| **Methods: Participants, interventions, and outcomes** | | |  |
| Trial setting | 13 | Settings (e.g., community, hospital) and locations (e.g., countries, sites) where the trial will be conducted | **Materials and Methods** (Study design), p.193 |
| Eligibility criteria | 14a | Eligibility criteria for participants | **Materials and Methods** (Participants), p.7 lines 198-214 |
|  | 14b | If applicable, eligibility criteria for sites and for individuals who will deliver the interventions (e.g., surgeons, physiotherapists) | Not Applicable |
| Intervention and comparator | 15a | Intervention and comparator with sufficient details to allow replication including how, when, and by whom they will be administered. If relevant, where additional materials describing the intervention and comparator (e.g., intervention manual) can be accessed | Materials and Methods (Intervention), p.8-10, lines 242-277 |
|  | 15b | Criteria for discontinuing or modifying allocated intervention/comparator for a trial participant (e.g., drug dose change in response to harms, participant request, or improving/worsening disease) | Not Applicable |
|  | 15c | Strategies to improve adherence to intervention/comparator protocols, if applicable, and any procedures for monitoring adherence (e.g., drug tablet return, sessions attended) | Not Applicable |
|  | 15d | Concomitant care that is permitted or prohibited during the trial | Not Applicable |
| Outcomes | 16 | Primary and secondary outcomes, including the specific measurement variable (e.g., systolic blood pressure), analysis metric (e.g., change from baseline, final value, time to event), method of aggregation (e.g., median, proportion), and time point for each outcome | **Materials and Methods** (Outcomes), p.10-12, lines 285-357 |
| Harms | 17 | How harms are defined and will be assessed (e.g., systematically, non-systematically) | **Materials and Methods** (Participants withdrawal and adverse events), p15, lines 442-459 |
| Participant timeline | 18 | Time schedule of enrollment, interventions (including any run-ins and washouts), assessments, and visits for participants. A schematic diagram is highly recommended (see Figure) | **Table 1** |
| Sample size | 19 | How sample size was determined, including all assumptions supporting the sample size calculation | **Materials and Methods** (Sample size calculation), p. 12-13, lines 359-369 |
| Recruitment | 20 | Strategies for achieving adequate participant enrollment to reach target sample size | **Materials and Methods** (Participants), p.7, lines 200-202 |
| **Methods: Assignment of interventions** | | |  |
| Randomization: |  |  |  |
| Sequence generation | 21a | Who will generate the random allocation sequence and the method used | **Materials and Methods** (Randomization/blinding), p. 8, lines 215-240 |
|  | 21b | Type of randomization (simple or restricted) and details of any factors for stratification. To reduce predictability of a random sequence, other details of any planned restriction (e.g., blocking) should be provided in a separate document that is unavailable to those who enroll participants or assign interventions | **Materials and Methods** (Randomization/blinding), p.8, lines 218-221 |
| Allocation concealment  mechanism | 22 | Mechanism used to implement the random allocation sequence (e.g., central computer/telephone; sequentially numbered, opaque, sealed containers), describing any steps to conceal the sequence until interventions are assigned | **Materials and Methods** (Randomization/blinding), p.8, lines 216-218 |
| Implementation | 23 | Whether the personnel who will enroll and those who will assign participants to the interventions will have access to the random allocation sequence | **Materials and Methods** (Randomization/blinding) p.8, lines 216-218 |
| Blinding | 24a | Who will be blinded after assignment to interventions (e.g., participants, care providers, outcome assessors, data analysts) | **Materials and Methods** (Randomization/blinding) p.8, lines 226-239 |
|  | 24b | If blinded, how blinding will be achieved and description of the similarity of interventions | Materials and Methods (Randomization/blinding and Intervention) p.8, lines 226-239 |
|  | 24c | If blinded, circumstances under which unblinding is permissible, and procedure for revealing a participant’s allocated intervention during the trial | Materials and Methods (Randomization/blinding) p.8, lines 239-240 |
| **Methods: Data collection, management, and analysis** | | |  |
| Data collection methods | 25a | Plans for assessment and collection of trial data, including any related processes to promote data quality (e.g., duplicate measurements, training of assessors) and a description of trial instruments (e.g., questionnaires, laboratory tests) along with their reliability and validity, if known. Reference to where data collection forms can be accessed, if not in the protocol | **Materials and Methods** (Outcomes)  p.10-12, lines 279-357 |
|  | 25b | Plans to promote participant retention and complete follow-up, including list of any outcome data to be collected for participants who discontinue or deviate from intervention protocols | **Materials and Methods**, p.14-15, lines 423-440 |
| Data management | 26 | Plans for data entry, coding, security, and storage, including any related processes to promote data quality (e.g., double data entry; range checks for data values). Reference to where details of data management procedures can be accessed, if not in the protocol | **Materials and Methods**, p.14-15, lines 423-440 |
| Statistical methods | 27a | Statistical methods used to compare groups for primary and secondary outcomes, including harms | **Materials and Methods** (Statistical analysis), p.13-14, lines 374-416 |
|  | 27b | Definition of who will be included in each analysis (e.g., all randomized participants), and in which group | **Materials and Methods**, p.14 line 400. |
|  | 27c | How missing data will be handled in the analysis | **Materials and Methods**, p.14 line 400. |
|  | 27d | Methods for any additional analyses (e.g., subgroup and sensitivity analyses) | Not Applicable |
| **Methods: Monitoring** | | |  |
| Data monitoring committee | 28a | Composition of data monitoring committee (DMC); summary of its role and reporting structure; statement of whether it is independent from the sponsor and funder; conflicts of interest and reference to where further details about its charter can be found, if not in the protocol. Alternatively, an explanation of why a DMC is not needed | Not Applicable |
|  | 28b | Explanation of any interim analyses and stopping guidelines, including who will have access to these interim results and make the final decision to terminate the trial | Not Applicable |
| Trial monitoring | 29 | Frequency and procedures for monitoring trial conduct. If there is no monitoring, give explanation | Not Applicable |
| **Ethics** | | |  |
| Research ethics approval | 30 | Plans for seeking research ethics committee/institutional review board approval | **Materials and Methods** (Ethics and dissemination), p 14-15, lines 423-470. |
| Protocol amendments | 31 | Plans for communicating important protocol modifications to relevant parties | **Materials and Methods** (Ethics and dissemination), p 14-15, lines 423-470. |
| Consent or assent | 32a | Who will obtain informed consent or assent from potential trial participants or authorized proxies, and how | **Materials and Methods** (Ethics and dissemination), p 14-15, lines 423-470. |
|  | 32b | Additional consent provisions for collection and use of participant data and biological specimens in ancillary studies, if applicable | Not applicable |
| Confidentiality | 33 | How personal information about potential and enrolled participants will be collected, shared, and maintained in order to protect confidentiality before, during, and after the trial | **Materials and Methods** (Ethics and dissemination), p 14-15, lines 423-427. |
| Ancillary and post-trial care | 34 | Provisions, if any, for ancillary and post-trial care, and for compensation to those who suffer harm from trial participation | Not applicable |

*We strongly recommend reading this checklist in conjunction with the SPIRIT 2025 Explanation and Elaboration and the SPIRIT 2025 Expanded Checklist for important clarifications on all the items. We also recommend reading relevant SPIRIT extensions. See [www.consort-spirit.org](http://www.consort-spirit.org)

Citation: Chan A-W, Boutron I, Hopewell S, Moher D, Schulz KF, et al. SPIRIT 2025 statement: updated guideline for protocols of randomised trials. BMJ 2025;389:e081477. <https://dx.doi.org/10.1136/bmj-2024-081477>

© 2025 Chan A-W et al. This is an Open Access article distributed under the terms of the Creative Commons Attribution License (<https://creativecommons.org/licenses/by/4.0/>), which permits unrestricted use, distribution, and reproduction in any medium, provided the original work is properly cited.
