# Supplementary material for: Transcranial direct current stimulation (tDCS) over the dorso-lateral-prefrontal cortex in combination with exercises for the treatment of individuals with chronic low back pain (STOP-Low Back Pain Trial): study protocol for a randomised controlled trial
Source: BMJ Open. 2026 Mar 18;16(3):e111649. doi: 10.1136/bmjopen-2025-111649 (PMC13007130; doi:10.1136/bmjopen-2025-111649)
Supplement: online supplemental file 2 [file bmjopen-16-3-s002.docx]

**Supplemental material 2: Detailed Inclusion and Exclusion Criteria**

Comprehensive list of inclusion and exclusion criteria used to determine participant eligibility for the study.

Participants must meet **all** of the following criteria to be eligible for inclusion.

**Inclusion Criteria**:

- ability to give informed consent,
- ability to follow protocol instructions,
- diagnosis of Non Specific Chronic Low Back Pain ≥12 weeks,
- low back pain with or without radiation to the knee,
- average pain of the previous week ≥ 3 on the VAS (Visual Analogue Scale),
- have sufficient cognitive ability to fill in the various questionnaires (Level B2 French).

Participants meeting **any** of the following criteria are excluded from participation.

**Exclusion Criteria**:

- herniectomy within the last 6 months,
- lumbar spinal surgery with material (e.g. prosthesis, spondylodesis),
- sensory or motor deficit of a lower limb,
- radiant pain in the lower limb beyond the knee,
- neuropathic pain (according to the dn4 questionnaire),
- diagnosis of an inflammatory rheumatic disease (e.g. rheumatoid arthritis, spondyloarthropathy),
- diagnosis of a chronic generalized pain syndromee of fibromyalgia,
- pregnancy,
- presence of neurological or neuropsychiatric disorders,
- have epilepsy or a recent or severe head injury,
- metal implant in the skull (excluding fillings),
- presence of a pacemaker,
- unhealed wound or skin disease on the skull (electrode contact area).

All eligibility criteria were assessed during the screening visit prior to randomisation.
