# Supplementary material for: Transcranial direct current stimulation (tDCS) over the dorso-lateral-prefrontal cortex in combination with exercises for the treatment of individuals with chronic low back pain (STOP-Low Back Pain Trial): study protocol for a randomised controlled trial
Source: BMJ Open. 2026 Mar 18;16(3):e111649. doi: 10.1136/bmjopen-2025-111649 (PMC13007130; doi:10.1136/bmjopen-2025-111649)
Supplement: online supplemental file 4 [file bmjopen-16-3-s004.docx]

**Supplementary material 4: Participant Information Sheet (French version)**

Hôpitaux Universitaires de Genève, Département de chirurgie, Division d'orthopédie et de chirurgie traumatologique, 1211 Genève.

Demande de participation à un projet de recherche médical :

**Stimulations cérébrales non-invasives innovantes dans la réhabilitation et la plasticité cérébrale chez le lombalgique chronique.**

Madame, Monsieur

Nous vous proposons de participer à notre projet de recherche.

Votre participation est entièrement libre. Toutes les données collectées dans le cadre de ce projet sont soumises à des règles strictes en matière de protection des données.

Le projet de recherche est mené par le Prof. Stéphane Armand et le Pr. Stéphane Genevay, Hôpitaux Universitaires de Genève (HUG). Nous vous en communiquerons les résultats si vous le souhaitez.

Lors d’un entretien, nous vous présenterons les éléments essentiels et répondrons à vos questions. Pour vous proposer d’ores et déjà un aperçu du projet, voici les points clés à retenir. Vous trouverez à la suite des informations complémentaires plus détaillées.

Pourquoi menons-nous ce projet de recherche ?

En présence de lombalgie chronique, on bénéficie de séances de physiothérapie, dans le but d’obtenir une diminution de la douleur ainsi qu’une amélioration de la capacité fonctionnelle et de la qualité de vie.

Notre projet de recherche vise à étudier si la combinaison d’applications de stimulations électriques cérébrales transcrâniennes (tDCS) non-invasives et de traitement de physiothérapie est plus efficace que le traitement de physiothérapie seul.

Que dois-je faire si j’accepte de participer ? – Que se passe-t-il pour moi en cas de participation ?

Forme de la participation :

Si vous acceptez de participer à notre projet, un médecin déterminera si vous répondez aux critères d’éligibilité de l’étude avant le début des visites. La signature d’un document attestant votre consentement à l’étude vous sera demandée. Vous répondrez à des questionnaires en lien avec votre maladie. Ensuite, vous aurez un électromyogramme des muscles du dos, c’est à dire des capteurs seront posés sur les muscles de votre dos pour mesurer leur activité pendant que vous vous penchez en avant. Ensuite, vous passerez un électroencephalogramme (EEG). Un casque sera posé sur votre tête pour enregistrer l’activité de votre cerveau au repos. Après ces examens vous participerez à 9 séances de physiothérapie et de stimulations crâniennes à l’hôpital de La Tour. Pendant ces séances, un casque avec des éponges sera posé sur votre tête pour stimuler une partie de votre cerveau (le cortex préfrontal) dans le but d’optimiser les effets de la séance de physiothérapie.

Déroulement pour les participant.e.s :

Les participant.e.s sont réparti.e.s au hasard dans 2 groupes : soit dans le groupe **Physiothérapie associée aux stimulations électriques transcrâniennes (tDCS) effectives** soit dans le groupe **Physiothérapie associée aux stimulations électriques (tDCS) placebo**. Le terme placebo signifie une stimulation comparable à l’autre groupe mais qui en réalité ne produit pas d’effet réel. Vous recevrez donc par l’intermédiaire d’un appareil de stimulation cérébrale transcrânienne appellé tDCS (transcranial Direct Current Stimulation) posé sur votre tête soit une stimulation effective soit une stimulation placebo en meme temps qu’un traitement de physiothérapie.

Durée :

Vous participerez aux sessions d’évaluations et de traitements pendant 5 semaines. Par la suite vous bénéficierez d’un suivi de votre état de santé par contact téléphonique ou e-mail à 3 et 6 mois après le début du programme.

Nombre de consultations, temps nécessaire et contraintes associées :

Une évaluation avant le traitement sera effectuée au laboratoire de neurorééducation des HUG ou au laboratoire de neuropsychologie d’UNI MAIL. L’évaluation durera environ 2h30 comprenant le remplissage de questionnaires, l’electromyographie (EMG) des muscles du dos et l’electroencéphalographie (EEG).

Les traitements se dérouleront à l’hôpital de La Tour à Meyrin. Ils comporteront 9 séances d’une heure au rythme de 3 fois par semaine pendant 3 semaines. Ces séances seront composées de physiothérapie active (exercices cardio-respiratoire, de renforcement, de contrôle moteur) combinée à un type de stimulation selon le groupe alloué.

Quels sont les bénéfices et les risques liés à la participation au projet ?

Bénéfices pour les participant.e.s

La participation à ce projet peut vous apporter l’avantage de bénéficier d’une nouvelle approche thérapeutique qui pourrait améliorer la douleur ressentie, ainsi que vos capacités physiques pouvant rendre plus facile la pratique d’activité physique ou de toute autre activité de la vie quotidienne et qui n’est actuellement pas offerte en dehors des protocoles de recherche. L’intervention (stimulations transcrâniennes) n’aura potentiellement un bénéfice que dans le groupe qui n’est pas placebo.

De plus vous bénéficierez d’une évaluation précise sur une durée de 6 mois.

Toutefois, aucune garantie de bénéfice ne peut être assurée.

Par votre participation, vous contribuez à aider d’autres personnes atteintes de la même maladie dans le futur et à ce traitement de devenir un standard dans les protocoles de réhabilitation.

Risques et contraintes

Les enregistrements des signaux biologiques ne sont pas invasifs et effectués avec des appareils disponibles sur le commerce et utilisés en routine dans l’hôpital : pour les enregistrements des signaux EEG et EMG, uniquement des électrodes de surface sont utilisées.

Les séances de stimulation tDCS comprennent l'utilisation d'un dispositif approuvé pour l'usage clinique et conforme au marquage CE. Son utilisation pourrait provoquer transitoirement des sensations de picotement, des démangeaisons ou de chaleur à l’endroit de la stimulation.

Pour l’enregistrement de l’activité cérébrale (EEG), vous allez porter un bonnet avec des électrodes de surface et du gel conducteur, qui sera positionné sur votre tête.

Les sessions de stimulation ou d’enregistrement pourraient vous fatiguer. Si c’est le cas, les procédures seront interrompues et vous serez libre de choisir de continuer ou de les interrompre.

En apposant votre signature à la fin du document, vous certifiez en avoir compris tout le contenu et consentir librement à prendre part au projet.

**Information détaillée**

1. **Objectif du projet et sélection des participant·e·s**

Dans cette feuille d’information, notre projet de recherche est aussi simplement désigné par le terme *étude*. Si vous acceptez d’y prendre part, vous êtes un.e *participant.e à l’étude*.

Avant qu’un dispositif médical soit employé, il faut l’évaluer scientifiquement chez des participant.e.s à une étude. Ce projet doit nous permettre d’examiner et de mesurer l’efficacité, la performance et la sécurité du dispositif médical de tDCS (stimulation transcrânienne par courant direct) dans le traitement de la lombalgie chronique. Cet appareil de tDCS présente les propriétés suivantes : il sert à moduler l’activité cérébrale en induisant, à travers le crâne, un faible courant électrique dans le cerveau afin d’améliorer le potentiel de récupération clinique. Cet appareil a déjà été évalué en recherche dans le traitement des douleurs chroniques. En effet, des investigations récentes ont montré que ce type de stimulation couplée aux thérapies traditionnelles telle que la physiothérapie peut être une option thérapeutique prometteuse.

Nous vous sollicitons car la participation est ouverte à toutes les personnes qui souffrent de lombalgie depuis plus de 3 mois.

1. **Informations générales sur le projet**

Nous savons encore peu de choses sur les effets de la tDCS stimulant le cortex préfrontal chez les personnes souffrant de lombalgie. Nous souhaitons donc découvrir si la combinaison de la stimulation par tDCS du cortex préfrontal aux exercices de physiothérapie apporte un bénéfice supérieur à une thérapie en physiothérapie seule auprès des patient.e.s souffrant de lombalgie chronique.

Si vous participez au projet, vous serez réparti.e au hasard (étude randomisée) soit dans le groupe **physiothérapie associé à des stimulations tDCS effectives** soit dans le groupe **physiothérapie associé à des stimulations tDCS placebo**.

Les 2 groupes effectuerons le même programme de physiothérapie, la seule différence entre les groupes sera le type de stimulation de l’appareil de tDCS qui sera **effective** ou **placebo**. Ni le thérapeute, ni les évaluateurs, ni vous (triple aveugle) ne saurez à quel groupe vous appartenez. Le terme placebo signifie une stimulation comparable aux autres groupes mais qui en réalité ne produit pas d’effet réel. On peut ainsi comparer et mieux évaluer si les améliorations observées sont réellement dues au dispositif étudié, autrement dit si celui-ci est réellement efficace (étude contrôlée contre placebo). Un collaborateur non impliqué dans l’étude sera responsable de la randomisation. Le choix du groupe alloué ne sera divulgué ni à vous, ni aux thérapeutes ni aux évaluateurs participant au protocole avant la fin du protocole. Les termes entre parenthèses sont expliqués dans le glossaire au point 15.

L’appareil de stimulation tDCS utilisé pour cette étude comporte un marquage CE. Cela veut dire que son utilisation est certifiée et conforme à la législation en vigueur en Suisse. De plus il sera utilisé conformément à son mode d’emploi. Cet appareil a déjà fait l’objet de recherche sur des patient.e.s souffrant de lombalgie chronique.

Durant chaque séance de traitement de physiothérapie, vous recevrez en même temps une stimulation par tDCS pendant 20 minutes. L’étude durera 5 semaines. C’est une étude monocentrique, c’est à dire qui se déroulera uniquement à Genève. Elle impliquera 48 patient.e.s lombalgiques. Le traitement de physiothérapie effectué lors de cette étude est un traitement standard répondant aux recommandations récentes de traitement de la lombalgie chronique.

Cette étude est réalisée dans le respect des prescriptions de la législation suisse. Nous suivons en outre l’ensemble des directives reconnues au niveau international. La commission d’éthique compétente a examiné et autorisé l’étude. Vous trouverez également un descriptif de l’étude sur le site Internet de l’Office fédéral de la santé publique : www.kofam.ch.

# **Déroulement pour les participant e s**

Pour cette étude vous serez sollicité pour 13 visites sur une période de 29 semaines. Toutefois, votre présence sera nécessaire lors de 5 semaines uniquement. En effet, les visites de suivi (visites 13 et 14) à 12 et 24 semaines après le début du traitement seront faites à distance (contact par mail ou téléphone).

**Visite 1** (sélection/inclusion) : La visite 1 (sélection des participant.e.s) va servir à déterminer si vous remplissez les critères d’inclusion/exclusion, à signer le document attestant votre consentement à l’étude et répondre à vos questions. L’investigateur procédera à votre inclusion dans un groupe de stimulation (effectif ou placebo). Le délai entre la visite de sélection/inclusion et la deuxième visite correspondant aux évaluations cliniques et physiologiques débutera au plutôt le jour suivant.

**Visites 2** (pré-évaluations) : Les visites détaillées ci-dessous comprendront un examen EMG (évaluations de l’activité des muscles du dos lorsque vous vous penchez en avant), un examen EEG (évaluation de l’activité cérébrale au repos) et les questionnaires. Ces examens seront effectués à l’hôpital de Beau-Séjour des HUG ou au laboratoire de neuropsychologie d’UNI MAIL.

Evaluation 1 - évaluations par questionnaires (environ 30 minutes) : Votre état de santé sera évalué à l'aide des échelles d'évaluations cliniques suivantes :

1. Le Core Outcome Mesures Index (COMI),
2. L’échelle Visuelle Analogique (EVA) moyen des 7 derniers jours,
3. L’Oswestry Disability Index (ODI),
4. L’Hospital Anxiety and Depression Scale (HADS),
5. Le Fear Avoidance Belief Questionnaire (FABQ),
6. Le Pain Catastrophizing Scale (PCS).

Un investigateur recueillera vos évaluations cliniques citées précédemment. En cas de question, il vous aidera à les compléter.

Evaluation 2 – évaluation EEG (environ 1 h) : Votre activité cérébrale au repos sera enregistrée par la méthode de l’électroencéphalographie (EEG) durant 10 minutes. L’investigateur disposera le matériel EEG sur votre tête, veillera au bon fonctionnement et à votre confort. Puis, l’investigateur procédera au retrait du dispositif. Il se chargera également du lavage de vos cheveux. Enfin, il s’assurera de l’absence d’effets secondaires.

Evaluation 3 – évaluation EMG (environ 45 min) : des capteurs seront placés sur les muscles de votre dos afin d’évaluer leur activité lorsque vous vous pencherez en avant puis vous relèverez. Cette manœuvre sera répétée plusieurs fois. Les capteurs seront ensuite retirés et nous nous assurerons de votre confort.

**Visites 3-11** (traitements) : Après les pré-évaluations, vous aurez 3 semaines de traitement avec 3 visites de stimulation par semaine. Les visites de simulation seront différentes en fonction de votre groupe de réhabilitation.

- **Physiothérapie associée à des stimulations tDCS effectives :** tDCS excitatrice + séances de réhabilitation
  - 9 séances de stimulation combinées à une séance de physiothérapie (durée d’une heure).
- **Physiothérapie associée à des stimulations tDCS placebo :** tDCS placebo + séances de réhabilitation
  - 9 séances de stimulation combinées à une séance de physiothérapie (durée d’une heure).

Les traitements (physiothérapie combinée à la tDCS) seront effectués à l’hôpital de La Tour à Meyrin. Pour les visites 3 à 11, le.la physiothérapeute responsable du traitement procédera à la pose de la tDCS. Il.elle s’assurera du bon fonctionnement de la tDCS et de votre confort. Ensuite, il.elle vous conduira en physiothérapie. Au début de la thérapie, le.la physiothérapeute démarrera la stimulation. En fin de traitement, le.la physiothérapeute retirera la tDCS et s’assurera de l’absence d’effets indésirables. Pour cela, un document formel indiquant si la séance s’est déroulée normalement sans effet secondaire sera rédigé.

Quel que soit le groupe alloué, un membre du personnel de l’étude viendra 15-20 minutes avant la séance de thérapie installer le dispositif tDCS sur le crâne. Chaque séance de physiothérapie durera une heure et comprendra un entrainement individualisé avec une participation active du.de.la patient.e. Elle sera menée par un.e physiothérapeute expérimenté.e dans la prise en charge des patient.e.s lombalgiques chroniques. Dans cette étude, le programme se composera de différentes stations comprenant : (1) 20 min d’exercices cardiorespiratoires (marche sur tapis, vélo…) (2) 10 min d’exercices de renforcement (3) 10 min d’exercices de contrôle moteur (4) 10 min d’étirement. La progression se fera par l’augmentation du nombre de répétitions de l’exercice ou l’augmentation de sa difficulté. Vous pourrez prendre des pauses à tout moment et un.e thérapeute vous suivra pendant la séance. A la fin de la séance, un membre du personnel viendra retirer le dispositif de tDCS. Il s’assurera de votre bien-être et de votre retour à domicile.

**Visites 12 (post-évaluations) :** à la fin des 3 semaines de traitement, il y aura une nouvelle session d’évaluation soit :

Evaluation 1 - évaluation par questionnaire (environ 30 minutes) : Votre état de santé sera évalué à l'aide des mêmes échelles d'évaluations cliniques que lors de la visite 1 (pré-évaluation). En cas de question, il vous aidera à les compléter.

Evaluation 2 – évaluation EEG (environ 1 h) : Votre activité cérébrale au repos sera enregistrée par la méthode de l’électroencéphalographie (EEG) durant 10 minutes. L’investigateur disposera le matériel EEG sur votre tête, veillera au bon fonctionnement et à votre confort. Puis, l’investigateur procédera au retrait du dispositif. Il se chargera également du lavage de vos cheveux. Enfin, il s’assurera de l’absence d’effets secondaires auprès du.de la participant.e.

Evaluation 3 – évaluation EMG (durée d’environ 30 minutes) : des capteurs seront placés sur les muscles de votre dos afin d’évaluer leur activité lorsque vous vous pencherez en avant puis vous relèverez. Cette manœuvre sera répétée plusieurs fois. Les capteurs seront ensuite retirés et nous nous assurerons de votre confort.

**Visites 13-14 (post-évaluations) :** à trois et six mois après l’inclusion dans l’étude, il y aura une session d’évaluation respective composée de :

Evaluation 4 et 5- évaluations par questionnaire (environ 30 minutes) : Votre état de santé sera évalué à l'aide des mêmes questionnaires que ceux remplis lors de la visite 1 (pré-clinique).

Un investigateur recueillera vos évaluations cliniques par mail. En cas de question, il vous aidera à les compléter par téléphone si nécessaire.

|  | Examens | Traitement | Durée | Semaine | Lieux |
| --- | --- | --- | --- | --- | --- |
| Visite 1 | Vérification critères d’inclusion |  | 15 min |  | Cabinet médecin recruteur ou à distance |
| Visite 2 | Questionnaires, EMG et EEG |  | 2h45 | 1 | Hôpital Beau-séjour-HUG ou au laboratoire de neuropsychologie d’UNI MAIL |
| Visite 3 |  | Physiothérapie combinée aux stimulations tDCS | 1h | 2 | Hôpital de La Tour-Meyrin |
| Visite 4 |  | Physiothérapie combinée aux stimulations tDCS | 1h | 2 | Hôpital de La Tour-Meyrin |
| Visite 5 |  | Physiothérapie combinée aux stimulations tDCS | 1h | 2 | Hôpital de La Tour-Meyrin |
| Visite 6 |  | Physiothérapie combinée aux stimulations tDCS | 1h | 3 | Hôpital de La Tour-Meyrin |
| Visite 7 |  | Physiothérapie combinée aux stimulations tDCS | 1h | 3 | Hôpital de La Tour-Meyrin |
| Visite 8 |  | Physiothérapie combinée aux stimulations tDCS | 1h | 3 | Hôpital de La Tour-Meyrin |
| Visite 9 |  | Physiothérapie combinée aux stimulations tDCS | 1h | 4 | Hôpital de La Tour-Meyrin |
| Visite 10 |  | Physiothérapie combinée aux stimulations tDCS | 1h | 4 | Hôpital de La Tour-Meyrin |
| Visite 11 |  | Physiothérapie combinée aux stimulations tDCS | 1h | 4 | Hôpital de La Tour-Meyrin |
| Visite 12 | Questionnaires, EMG et EEG |  | 2h45 | 5 | Hôpital Beau-séjour-HUG ou au laboratoire de neuropsychologie d’UNI MAIL |
| Visite 13 | Questionnaires |  | 30 min | 12 | à distance (email ou téléphone) |
| Visite 14 | Questionnaires |  | 30 min | 24 | à distance (email ou téléphone) |

Il se peut que nous devions vous retirer de l’étude avant le terme prévu. Cette situation peut se produire si vous étiez confronté à un événement indésirable, une grossesse ou la survenue d’un problème de santé ne vous permettant plus de poursuivre l’étude. Dans ce cas, nous vous proposerons pour votre propre sécurité de vous examiner une dernière fois. La poursuite de votre prise en charge médical(e) est assurée en tout temps.

Votre médecin traitant·e sera informé·e de votre participation à cette étude.

# **Bénéfices pour les participant**·**e** ·s

Participer à cet essai clinique pourrait présenter pour vous l’avantage de bénéficier d’une nouvelle approche thérapeutique qui pourrait améliorer votre récupération clinique et qui n’est actuellement pas disponible en dehors des protocoles de recherche. De plus, vous bénéficierez d’une évaluation précise sur une durée de 6 mois.

Le bénéfice de cette nouvelle approche thérapeutique n’est cependant pas garanti ; il est possible que vous n’en tiriez aucun bénéfice.

De plus, votre participation pourrait permettre à cette approche thérapeutique de devenir un standard dans les protocoles de réhabilitation, afin d’aider d’autres personnes atteintes de la même pathologie que vous.

# **Caractère facultatif de la participation et obligations**

Votre participation est entièrement libre. Si vous choisissez de ne pas participer ou si vous choisissez de participer et revenez sur votre décision pendant le déroulement de l’étude, vous n’aurez pas à vous justifier. Cela ne changera rien à votre prise en charge médicale habituelle.

Si vous choisissez de participer à l’étude, vous serez tenu·e :

- de suivre les instructions de votre investigateur et de vous conformer au plan de l’étude prévu par le protocole de recherche, en particulier de suivre le programme et l’emploi du temps des séances de physiothérapie et de ne pas participer à une autre étude pendant toute la durée de celle-ci.
- d’informer l’investigateur et le médecin-investigateur de l’évolution de la maladie et de lui signaler tout nouveau symptôme, tout nouveau trouble et tout changement dans votre état ;
- d’informer le médecin-investigateur de tout traitement ou thérapie prescrit par une autre ou un autre médecin ainsi que de tous les médicaments que vous prenez y compris les médicaments de médecine complémentaire ou alternative.

# **Risques et contraintes pour les participant**·**e**·**s**

Les enregistrements des signaux biologiques ne sont pas invasifs et effectués avec des appareils disponibles sur le commerce et utilisés en routine dans l’hôpital : pour les enregistrements des signaux EEG et EMG, uniquement des électrodes de surface sont utilisées.

Les séances de stimulation tDCS comprennent l'utilisation d'un dispositif approuvé pour l'usage clinique et conforme au marquage **CE**. Son utilisation pourrait provoquer transitoirement des sensations de picotement, des démangeaisons ou de chaleur à l’endroit de la stimulation.

Pour l’enregistrement de l’activité cérébrale (EEG), vous allez porter un bonnet avec des électrodes de surface et du gel conducteur, qui sera positionné sur votre tête. Les sessions de stimulation ou d’enregistrement pourraient vous fatiguer. Si c’est le cas, les procédures seront interrompues et vous serez libre de choisir de continuer ou de les interrompre.

**Pour les femmes en état de procréer**

Les femmes enceintes sont formellement exclues de l’étude. Toutefois, si vous découvriez que vous êtes enceinte pendant l’étude, vous devez en informer immédiatement l’investigateur et le cas échéant être exclue de l’étude. Dans ce cas, vous serez priée de donner des informations sur l’évolution et l’issue de la grossesse.

# **Alternatives**

La participation à l’étude présente des bénéfices et des risques. Le programme de physiothérapie de cette étude répond aux recommandations de traitement thérapeutique de la lombalgie. D’autres traitements peuvent être recommandés pour cette pathologie comme les thérapies manuelles (massages, manipulations…), des programmes d’éducation thérapeutique ou de thérapie cognitivo-comportementales. La médecin-investigatrice ou le médecin-investigateur vous conseillera à ce sujet lors de l’entretien.

# **Résultats**

L’étude permet d’obtenir différents résultats :

1. des résultats individuels qui vous concernent directement,
2. les résultats définitifs objectifs de l’étude dans son ensemble.

1. Le médecin-investigateur vous avisera pendant l’étude de toute nouvelle découverte importante vous concernant. Vous serez informé.e oralement et par écrit ; vous pourrez par la suite à nouveau décider si vous souhaitez poursuivre votre participation à l’étude.

2. Le médecin-investigateur peut vous faire parvenir, à l’issue de l’étude, une synthèse des résultats globaux.

# **Confidentialité des données**

- 1. **Traitement et codage des données**

Dans le cadre de cette étude, des données relatives à votre personne et à votre santé sont recueillies et traitées, en partie de manière automatisée. Ces informations sont codées au moment du relevé. Le codage signifie que toutes les données permettant de vous identifier (nom, date de naissance, etc.) sont remplacées par un code. Il n’est pas possible de relier les données à votre personne sans le code, qui reste en permanence au sein de l’hôpital.

Seul un nombre limité de personnes peut consulter vos données sous une forme non codée, et ce, exclusivement afin de pouvoir accomplir des tâches nécessaires au déroulement de l’étude. Ces personnes sont tenues au secret professionnel. En tant que participant·e, vous avez le droit de consulter vos données.

- 1. **Protection des données**

Toutes les directives relatives à la protection des données sont rigoureusement respectées. Il est possible que vos données doivent être transmises sous forme codée, par exemple pour une publication, et qu'elles puissent être mises à la disposition d'autres chercheurs.ses.

- 1. **Droit de consultation dans le cadre d’inspections**

L’étude peut faire l’objet d’inspections. Celles-ci peuvent être effectuées par la commission d’éthique compétente, ou par le promoteur qui a initié l’étude. Le médecin-investigateur doit alors communiquer vos données pour les besoins de ces inspections. Toutes les personnes impliquées sont tenues au plus strict secret professionnel.

# **Retrait du projet**

Vous pouvez à tout moment vous retirer de l’étude si vous le souhaitez. Cependant, les données médicales et les données issues des évaluations recueillies jusque-là pourront encore être analysées sous forme codée.

En cas de retrait, vos données continuent de figurer sous forme codée dans les documents de l’étude, en premier lieu pour assurer la sécurité médicale. Vous devez donc être d’accord avec cela avant de donner votre consentement.

# **Dédommagement**

Vous percevrez la somme de 20 CHF lors de chacune des 2 évaluations conduites à l’hôpital Beau-séjour ou au laboratoire de neuropsychologie d’UNI MAIL (visite 2 et 12), soit une somme de 40 CHF au total si vous poursuivez jusqu’à la visite 12. Les examens qui font partie de l’étude n’occasionneront aucun frais additionnel.

# **Responsabilité**

La division d'orthopédie et de chirurgie traumatologique du Département de chirurgie des Hôpitaux Universitaires de Genève, qui a initié le projet de recherche et est chargée de sa réalisation, est responsable des dommages que vous pourriez subir en relation avec le dispositif à l’étude ou avec les activités de recherche (p. ex. examens). Les conditions et la procédure sont fixées par la loi.

Bien que ces recherches ne présentent pas de risque prévisible, l’institution (le promoteur) est responsable, en vertu des dispositions légales, de tout dommage pouvant survenir dans le cadre de l’étude.

Pour les dommages occasionnés par le dispositif médical de tDCS certifié et employé selon les instructions ou qui seraient survenus lors d’un traitement avec une thérapie conventionnelle, les règles de responsabilité applicables sont celles régissant les traitements en dehors d’une étude.

Si vous subissiez un dommage du fait de votre participation à l’étude, il vous faudrait vous adresser au médecin-investigateur.

# **Financement**

L’étude est majoritairement financée par le laboratoire de cinésiologie des HUG.L’appareil de tDCS est mis à disposition gratuitement par le service de cinésiologie des HUG, le personnel de physiothérapie menant l’intervention à l’hôpital de La Tour est rémunéré par cette institution partenaire du projet.

# **Interlocuteur(s)**

Vous pouvez à tout moment poser des questions au sujet de l’étude. En cas de doutes, de craintes ou d’urgences pendant ou après l’étude, vous pouvez vous adresser à l’un des interlocuteurs suivants :

Nom de l’investigateur principal :

**Pr. Stéphane ARMAND**

Institution: Hôpitaux Universitaire de Genève

Laboratoire de cinésiologie Willy Taillard

Email: stephane.armand@hcuge.ch

Téléphone: +41 22 37 **27 827**

Co-Investigateur :

Thomas Pourchet

Assistant filière physiothérapie

Haute école de santé / HES-SO Genève

Adresse : 25, rue des Caroubiers 1227 Carouge

Téléphone : +41 22 558 66 83

E-Mail : [thomas.pourchet@hesge.ch](mailto:thomas.pourchet@hesge.ch)

# Glossaire (termes nécessitant une explication)

- Placebo

Certaines personnes à qui on donne une préparation testée / un médicament ne recouvrent pas la santé grâce à cette préparation / ce médicament mais grâce au bien que leur procure l’attention de leur médecin. On se rend bien compte de ce phénomène quand on voit certains patients guérir alors qu’on leur a remis un pseudo-médicament, c’est-à-dire qui a l’apparence d’un vrai médicament, qui présente le même emballage, mais qui ne contient en réalité aucun principe actif. C’est ce type de pseudo-médicament qu’on appelle « placebo ».
Lors d’un essai clinique, il arrive qu’on décide de traiter une partie des participants avec le vrai médicament (contenant le principe actif) et une autre partie avec un placebo (sans principe actif). On peut ainsi comparer et mieux évaluer si les améliorations observées sont réellement dues à la préparation / au médicament étudié(e), autrement dit si celle-ci / celui-ci est réellement efficace, ou si ces améliorations sont à mettre sur le compte de l’attention accordée au patient ou à l’évolution naturelle de la maladie.

- Randomisé

Beaucoup d’études consistent à comparer deux ou plusieurs types de traitements différents. On peut, par exemple, comparer un traitement par la préparation testée / un médicament réel et un « traitement » par placebo. Il faut alors former deux groupes de participants, à savoir un groupe qui reçoit la préparation testée / le vrai médicament et un autre qui reçoit le placebo. Le terme « randomiser » désigne le fait de déterminer par tirage au sort le groupe dans lequel chaque participant sera. C’est donc le hasard qui décide si tel ou tel participant reçoit la préparation testée / le vrai médicament ou le placebo.

- Simple aveugle et double aveugle
- La procédure en simple ou double aveugle permet d’obtenir des résultats plus précis et plus fiables. On parle d’étude en simple aveugle lorsque les participants **ou** les chercheurs ne savent pas qui reçoit la préparation testée / le vrai médicament et qui reçoit le placebo. La répartition des participants en groupes se fait au moyen d’un tirage au sort, que les chercheurs confient à une personne extérieure à l’étude.

On parle d’étude en double aveugle lorsque **ni** les participants, **ni** les chercheurs ne savent qui reçoit la préparation testée / le vrai médicament et qui reçoit le placebo. La personne qui a effectué le tirage au sort ne lève normalement le secret sur la composition des groupes qu’une fois l’étude terminée. Mais elle peut le faire à tout moment si la situation l’exige.

Une personne qui sait qu’elle reçoit la préparation testée / le vrai médicament est beaucoup plus attentive aux réactions de son corps qu’une personne qui sait qu’elle ne reçoit que le placebo. Cela peut la conduire à surestimer l’efficacité de la préparation testée / du médicament.

- Étude randomisée, en double aveugle, contrôlée par placebo

L’étude vise à évaluer l’efficacité de la préparation testée / du médicament. Pour ce faire, les participants sont (généralement) divisés en deux groupes. Ceux du premier groupe reçoivent la préparation / le médicament à évaluer. Ceux du second groupe reçoivent un placebo, c’est-à-dire un pseudo-médicament qui aura l’apparence du vrai mais ne contiendra en réalité aucun principe actif. On peut alors savoir…

- Promoteur

Le promoteur est une personne ou une institution ayant son siège ou une représentation en Suisse qui prend l’initiative d’une étude, c.-à-d. qui porte la responsabilité de son lancement, de sa gestion et de son financement dans ce pays.

**Déclaration de consentement**

**Déclaration de consentement écrite pour la participation à une étude clinique**

Veuillez lire attentivement ce formulaire. N’hésitez pas à poser des questions lorsque vous ne comprenez pas quelque chose ou que vous souhaitez avoir des précisions. Votre consentement écrit est nécessaire pour participer au projet.

| **Numéro BASEC du projet de recherche (après soumission à la commission d’éthique compétente) :** | 2022.d0077 |
| --- | --- |
| **Titre (scientifique et usuel) :** | Stimulation Transcrânienne d’Optimisation en Physiothérapie – Lombalgie Chronique  STOP - Lombalgie |
| **Institution responsable (promoteur et adresse complète) :** | Hôpitaux Universitaires de Genève, Département de chirurgie, Division d'orthopédie et de chirurgie traumatologique, 1211 Genève. |
| **Lieu de réalisation :** |  |
| **Investigateur ou co-investigateur responsable sur le site :** Nom et prénom en caractères d’imprimerie : |  |
| **Participant / Participante :** Nom et prénom en caractères d’imprimerie : Date de naissance : |  |

- Je déclare avoir été informé·e, par le médecin-investigateur soussigné, oralement et par écrit, des objectifs et du déroulement de l’étude mettant en œuvre le dispositif médical de tDCS ainsi que des avantages et des inconvénients possibles et des risques éventuels.
- Je prends part à cette étude de façon volontaire et j’accepte le contenu de la feuille d’information qui m’a été remise. J’ai eu suffisamment de temps pour prendre ma décision.
- J’ai reçu les réponses aux questions que j’ai posées en relation avec ma participation à cette étude. Je conserve la feuille d’information et reçois une copie de ma déclaration de consentement écrite.
- J’ai été informé·e des alternatives thérapeutiques au projet, p. ex. de l’existence d’autres traitements et thérapies.
- J’accepte que ma médecin traitante/mon médecin traitant soit informé·e de ma participation à l’étude.
- J’accepte que les spécialistes compétents du promoteur de ce projet, de la commission d’éthique compétente puissent consulter mes données non codées afin de procéder à des contrôles et des inspections, à condition toutefois que la confidentialité de ces données soit strictement assurée.
- Je serai informé·e des résultats ayant une incidence directe sur ma santé. Si je ne souhaite pas obtenir ces informations, je prends contact avec la médecin-investigatrice / le médecin-investigateur.
- Je sais que mes données personnelles, mes données de santé (et mes échantillons) peuvent être transmis(es) à des fins de recherche dans le cadre de cette étude et uniquement sous une forme codée. Le promoteur assure une protection des données conforme aux normes et exigences suisses.
- Je peux, à tout moment et sans avoir à me justifier, révoquer mon consentement à participer à l’étude, sans que cette décision n’ait de répercussions défavorables sur la suite de ma prise en charge. Les données et le matériel biologique qui ont été recueillis jusque-là seront cependant analysés dans le cadre de l’étude.
- Je suis informé·e que l’assurance-responsabilité civile de l’hôpital / l’institution couvre les dommages éventuels imputables au projet.
- Je suis conscient·e que les obligations mentionnées dans la feuille d’information destinée aux participant·e·s doivent être respectées pendant toute la durée de l’étude. L’investigateur /le co-investigateur peut m’exclure à tout moment de l’étude dans l’intérêt de ma santé.

| Lieu, date | Signature du participant / de la participante |
| --- | --- |

**Attestation de l’investigateur / du co-investigateur :** Par la présente, j’atteste avoir expliqué au participant / à la participante la nature, l’importance et la portée de l’étude. Je déclare satisfaire à toutes les obligations en relation avec cette étude conformément au droit suisse en vigueur. Si je devais prendre connaissance, à quelque moment que ce soit durant la réalisation de l’étude, d’éléments susceptibles d’influer sur le consentement du participant / de la participante à prendre part au projet, je m’engage à l’en informer immédiatement.

| Lieu, date | Nom et prénom la médecine-investigatrice / du médecin-investigateur en caractères d’imprimerie.  Signature de l’investigateur / du co-investigateur |
| --- | --- |
