# Supplementary material for: Transcranial direct current stimulation (tDCS) over the dorso-lateral-prefrontal cortex in combination with exercises for the treatment of individuals with chronic low back pain (STOP-Low Back Pain Trial): study protocol for a randomised controlled trial
Source: BMJ Open. 2026 Mar 18;16(3):e111649. doi: 10.1136/bmjopen-2025-111649 (PMC13007130; doi:10.1136/bmjopen-2025-111649)
Supplement: Supplementary file 5 [file bmjopen-2025-111649.reviewer_comments.pdf]

PEER REVIEW HISTORY

BMJ Open publishes all reviews undertaken for accepted manuscripts. Reviewers are asked to complete a checklist review form and are provided with free text boxes to elaborate on their assessment. These free text comments are reproduced below.

ARTICLE DETAILS

Title (Provisional)

Transcranial Direct Current Stimulation (tDCS) over the dorso-lateral-prefrontal cortex in combination with exercises for the treatment of individuals with chronic low back pain (STOP-Low back pain Trial): study protocol for a randomized controlled trial

Authors

Pourchet, Thomas; Armand, Stéphane; Martins, Cristiano; Barrué-Belou, Simon; Genevay, Stéphane; Nicolo, Pierre

---

VERSION 1 - REVIEW

**Reviewer**	**1**
**Name**	**Hansford, Harrison**
**Affiliation**	**University of New South Wales, School of Health Sciences**
**Date**	**08-Dec-2025**
**COI**	**None**

The authors present a protocol for a randomised controlled trial of Transcranial Direct Current Stimulation (tDCS) and exercise compared to sham tDCS and exercise. The protocol addresses an important clinical question and is based on a sound study concept. The aims are relevant and the proposed design appears appropriate to answering the research objectives. The current version of the protocol lacks sufficient detail in key areas, which would need to be addressed before publication.

Abstract

Page 3, Lines 53-55: Please include the comparator group when describing the aim of the study.

Introduction

Page 5, Line 106: A minor suggestion to improve the sentence. The end of this sentence should be amended to read: “remains to be explored.”

Methods

Page 7, Line 165: There is a spelling error in the title “Materials and Methods:”

Page 7, Line 168: Please keep the language consistent between the terms sham and placebo control throughout the protocol. The following reference provides recommendations for reporting control groups https://doi.org/10.1136/bmj-2022-072108

Page 7, Line 169: Please specify whether participants will be blinded to their treatment allocation here.

Page 8, Line 181-182: Please report all aspects of this protocol as per the SPIRIT 2025 guidelines and reference the guidelines https://www.bmj.com/content/389/bmj-2024-081477. Please upload a completed checklist in the supplementary material.

Page 8, Line 194-195: Please reference this questionnaire. Consider https://pubmed.ncbi.nlm.nih.gov/38531755/ or 10.1016/j.pain.2004.12.010

Page 8, Line 198: Please describe whether contraindications to exercise were also included at eligibility assessment and if so, what these were.

Page 8, Line 200: Please state the full list of tDCS (+/- exercise) contraindications in a list in a supplement or specify these in the text.

Page 9, Lines 211-212: Please clarify this sentence i.e. blinded to … treatment allocation?

Page 9, line 212: Please provide more information on the outcome assessors, if the patients are completing patient-reported outcomes then they are the outcome assessors. Who else will be collecting outcomes?

Page 9, Line 240-241: Please provide more information on the device used – is it the same device with different modes?

Page 10, Lines 250-251: Please provide more information (or a reference) for the Borg scale used to provide clarity for the reader, i.e., is it the 0-10 or 6-20 scale?

Page 10, Lines 252-253: Please provide more information on the exercise protocol. Was this the same for each participant or changed based on participant capacity?

Page 10, Line 257: Please provide more detail on each scale used to orient the reader.

Page 10, Line 258: Please reference the SPIRIT guideline. Please ensure the whole protocol adheres to these guidelines as mentioned in an earlier comment.

Page 14, Line 367-368: Please reference this approach or consider use of established approaches to adjust for multiplicity (e.g., Bonferroni correction)

Page 15, Line 419-421: This approach may require some further though to ensure all adverse events are appropriately captured. See the following reference: https://pubmed.ncbi.nlm.nih.gov/21320389/

Page 16, Line 456: The protocol is described as rigorous and controlled, whilst the tDCS protocol is controlled the exercise protocol does not have enough detail to be considered for readers to assess this. Please provide more detail on the exercise component to clarify this.

Page 17, Line 484-485: Here is a statement referring to PPI described in the methods section. I was unable to see details about this, please include in this statement details at which stages patients were involved in and what their specific contributions were.

Review completed by Ms Martjie Venter and Mr Harrison Hansford

---

VERSION 1 - AUTHOR RESPONSE

Reviewer: 1

C: ***Abstract*** *Page 3, Lines 53-55: Please include the comparator group when describing the aim of the study.*

R: Thank you for this comment. The abstract has been revised to explicitly include the comparator group when describing the aim of the study (pages 2, lines 61-63). The objective now clearly states a comparison between anodal dorsolateral prefrontal cortex (DLPFC) tDCS combined with a standardised exercise programme and sham DLPFC tDCS combined with the same exercise programme on pain and function in adults with chronic low back pain.

C: ***Introduction*** *Page 5, Line 106: A minor suggestion to improve the sentence. The end of this sentence should be amended to read: "remains to be explored."*

R: Thank you for this suggestion. The sentence has been revised as recommended, and the wording now ends with "remains to be explored"(page 4, Line 118).

C: ***Methods*** *Page 7, Line 165: There is a spelling error in the title "Materials and Methods:"*

R: Thank you for pointing this out. The spelling error in the section title "Materials and Methods" has been corrected (page 6, line 176).

C: ***Methods*** *Page 7, Line 168: Please keep the language consistent between the terms sham and placebo control throughout the protocol. The following reference provides recommendations for reporting control groups https://doi.org/10.1136/bmj-2022-072108:.*

R: Thank you for this comment. We have ensured consistent terminology throughout the manuscript by **retaining the term "sham"** to accurately reflect the nature of the comparator intervention, rather than a usual-care or no-treatment control. This terminology was chosen in line with current recommendations for reporting control groups in non-invasive brain stimulation trials, as referenced.

C: ***Methods*** *Page 7, Line 169: Please specify whether participants will be blinded to their treatment allocation here.*

R: Thank you for this comment. The manuscript has been revised to clearly specify that participants will be blinded to their treatment allocation, in line with the study's blinding procedures (page 6, line 180).

C: ***Methods*** *Page 8, Line 181-182: Please report all aspects of this protocol as per the SPIRIT 2025 guidelines and reference the guidelines https://www.bmj.com/content/389/bmj-2024-081477. Please upload a completed checklist in the supplementary material. /* ***Methods*** *Page 8, Line 194-195: Please reference this questionnaire. Consider https://pubmed.ncbi.nlm.nih.gov/38531755/ or 10.1016/j.pain.2004.12.010*

R: Thank you for these comments. The protocol has been reviewed to ensure full compliance with the SPIRIT 2025 guidelines, which are now explicitly referenced in the manuscript (page 7, lines 194–195). In addition, a completed SPIRIT 2025 checklist, indicating the corresponding page and line numbers for each item, has been uploaded as supplementary material.

Furthermore, the questionnaire used in this study is now appropriately referenced in the manuscript, in accordance with the suggested sources.

C: ***Methods*** *Page 8, Line 198: Please describe whether contraindications to exercise were also included at eligibility assessment and if so, what these were.*

R: Thank you for this comment. No specific exercise-related contraindications were applied at eligibility assessment beyond the general exclusion criteria described in the manuscript. Participants were screened to exclude specific causes of low back pain, neurological deficits, and other medical conditions that could compromise safe participation. All exercise sessions are supervised by a licensed physiotherapist, and the exercises are individually adapted according to participants' symptoms and tolerance to ensure safety (page 7, lines 213-214)..

C: ***Methods*** *Page 8, Line 200: Please state the full list of tDCS (+/- exercise) contraindications in a list in a supplement or specify these in the text.*

R: Thank you for this comment. The full list of tDCS (+/− exercise) contraindications has now been explicitly detailed and provided as **supplementary material 2**, as requested (page 7, line 214).

C: ***Methods*** *Page 9, Lines 211-212: Please clarify this sentence i.e. blinded to … treatment allocation?*

R: Thank you for this comment. The sentence has been revised to clearly specify that blinding refers to **treatment allocation** (page 8, line 227-231).

C: ***Methods*** *Page 9, line 212: Please provide more information on the outcome assessors, if the patients are completing patient-reported outcomes then they are the outcome assessors. Who else will be collecting outcomes?*

R: Thank you for this comment. The manuscript has been revised to clarify the role of outcome assessors. It is now specified that patient-reported outcomes are completed by the participants themselves, while objective outcomes are collected by trained assessors who are blinded to treatment allocation (page 10, lines 281-283).

C: ***Methods*** *Page 9, Line 240-241: Please provide more information on the device used – is it the same device with different modes?*

R: Thank you for this comment. The manuscript has been revised to clarify that the same tDCS device is used across all study groups, with different stimulation modes applied according to treatment allocation (page 9, lines 256-258).

C: ***Methods*** *Page 10, Lines 250-251: Please provide more information (or a reference) for the Borg scale used to provide clarity for the reader, i.e., is it the 0-10 or 6-20 scale?*

R: Thank you for this comment. The manuscript has been revised to specify that the Borg Rating of Perceived Exertion 0–10 scale is used, and an appropriate reference has been added to provide clarity for the reader (page 9, lines 268-269).

C: ***Methods*** *Page 10, Lines 252-253: Please provide more information on the exercise protocol. Was this the same for each participant or changed based on participant capacity?*

R: Thank you for this comment. A detailed description of the exercise protocol has been added as **supplementary material 3**. The manuscript now clarifies that the exercise programme is standardised in its structure, while being individually adapted according to each participant's capacity, symptoms, and tolerance (page 9, line 277).

C: ***Methods*** *Page 10, Line 257: Please provide more detail on each scale used to orient the reader.*

R: Thank you for this comment. Additional details have been added for each scale used in the study to better orient the reader (pages 10, lines 280–283).

C: ***Methods*** *Page 10, Line 258: Please reference the SPIRIT guideline. Please ensure the whole protocol adheres to these guidelines as mentioned in an earlier comment.*

R: Thank you for this comment. The SPIRIT guidelines are now explicitly referenced in the manuscript (page 7, line 194-195). The protocol has been reviewed to ensure full adherence to the SPIRIT recommendations, and a completed SPIRIT checklist has been provided as **supplementary material 1**, as previously indicated.

C: ***Methods*** *Page 14, Line 367-368: Please reference this approach or consider use of established approaches to adjust for multiplicity (e.g., Bonferroni correction)*

R: Thank you for this comment. The statistical approach used to address multiplicity has now been clarified and appropriately referenced in the manuscript (page 13, line 389). Specifically, we refer to an established rule-of-thumb for multivariable linear regression models to justify the analytical strategy. This approach was defined a priori and developed in consultation with a statistician, rather than applying post-hoc multiplicity corrections such as Bonferroni.

C: ***Discussion*** *Page 15, Line 419-421: This approach may require some further though to ensure all adverse events are appropriately captured. See the following reference: https://pubmed.ncbi.nlm.nih.gov/21320389/*

R: Thank you for this comment. We acknowledge the reviewer's concern regarding the comprehensive capture of adverse events and appreciate the referenced guidance. As this manuscript reports a pre-registered study protocol, the adverse event monitoring procedures could not be modified *a posteriori*. However, this issue has now been explicitly acknowledged as a limitation in the manuscript, and the recommended

reference has been cited to appropriately contextualise this point (page 16, lines 485-489).

C: ***Discussion*** *Page 16, Line 456: The protocol is described as rigorous and controlled, whilst the tDCS protocol is controlled the exercise protocol does not have enough detail to be considered for readers to assess this. Please provide more details on the exercise component to clarify this.*

R: Thank you for this comment. A detailed description of the exercise protocol has now been provided as **supplementary material 3**, enabling readers to fully assess the rigour, standardisation, and reproducibility of the exercise component alongside the controlled tDCS protocol. The manuscript has been updated accordingly to direct readers to this supplementary material.

C: ***Discussion*** *Page 17, Line 484-485: Here is a statement referring to PPI described in the methods section. I was unable to see details about this, please include in this statement details at which stages patients were involved in and what their specific contributions were.*

R: Thank you for highlighting this point. We acknowledge that the original statement was inaccurate. The manuscript has been corrected to clearly state that patients and/or the public were not involved in the design, conduct, reporting, or dissemination of this research, in accordance with BMJ Open reporting require (page 17, lines 508-510).

VERSION 2 - REVIEW

**Reviewer**	**1**
**Name**	**Hansford, Harrison**
**Affiliation**	**University of New South Wales, School of Health Sciences**
**Date**	**20-Feb-2026**
**COI**	

Thank you for adequately responding to all of our comments, and reporting the protocol consistent with SPIRIT, the protocol is much improved. Best of luck in the conduct of this trial.
